# Notes on Celluloid

**Published:** 1875-05

**Authors:** J. B. Hodgkin

**Affiliations:** Professor of Dental Mechanism and Metallurgy, Baltimore College of Dental Surgery.


### AMERICAN JOURNAL

OF

### DENTAL SCIENCE.

Vol. IX. THIRD SERIES—MAY, 1875. No. 1.

### ARTICLE I.

### *Notes on Celluloid.*

BY J. B. HODGKIN, D. D. S.

Professor of Dental Mechanism and Metallurgy, Baltimore College of Dental Surgery.

The comparative perfection to which this base for artificial teeth has been brought is a matter of congratulation on the part of those of the Dental profession who have been looking to it as a relief from the vulcanite monopoly, or in the expectation of finding in it something intrinsically better than the rubber plates lately so universally made and worn. The beauty of the material, its elegant appearance, its near approach to a gum color, are really advantages so great as to tempt one to overlook any defects that may have been found in its working qualities or in its durability. The former difficulty, it would seem, has been pretty effectually overcome, and the latter, it is hoped, will be found by the grand test of time to be at least equal to the substance it is the rival of—vulcanite. It is worth while to say in passing, that the durability of vulcanite plates seems to have been over-estimated by many, and that a fair average of their life is not probably over five or six years. While a few do last much longer, yet the large majority of the plates that are made soon break down.

The advantages of celluloid over vulcanite, so far as regards the question of mercurial poisoning, are manifest, and need not be more than adverted to. Whether our friends, the homœopaths, will be able to make cases of camphor poisoning out of it, remains to be seen.

Possibly some better solvent, and one in which the solvent itself may enter more solidly into a combination with the cellulose may yet be found,—one possessing the properties of ether, without its objectionable qualities. That we are only on the threshold of discoveries in this field seems highly probable.

It is not likely that the best method of working this substance has yet been evolved, for like all things else, these matters seem to grow slowly, and our knowledge and wisdom do not always go hand in hand. With regard to the preparation of the mould, if the method devised by Dr. McClelland, as applied by him to Rose Pearl, which is only another form of celluloid, were rid of its undesirable features, it would possess advantages over the plaster moulds in ordinary use, until at least good dental plaster becomes the rule and not the exception; for with these metallic moulds a smoothness and finish can be given to the plate which leaves nothing to be done on removing it from the press except to cut away the surplus and polish the edges thus cut. Plaster moulds, however, may, with sufficient care, be made so smooth as to require no finishing, as the celluloid does not sink into the surface of the plaster, as does vulcanite. In cases lately constructed by the writer, the finish of the piece as it came from the flask could not well be improved; plain teeth being used and the wax surface smoothed with the blow-pipe. The gum color was sufficiently good to answer the purpose, and the fact that plain teeth are used, allows an artistic arrangement rarely attempted and not often practicable with block gum sections·

The best apparatus for softening the plate and closing the flask is probably yet to be constructed, but of those so far made all seem in careful hands to produce good results. Few, however, would willingly adhere to the "oil" method, which we believe was the first practiced, with its suggestions of "lard rendering, all over the house, if a more pleasant and equally efficacious plan were presented; and fortunately in celluloid we have quite a variety of methods from which to choose. Glycerin or steam are better than oil in the sense of being more pleasant and agreeable, and the dry process as worked in several different forms is still better in not having any liquids to handle, and in not incurring (as in the steam apparatus, at least,) the suspicion of a risk of an explosion.

Several forms of the "dry process" are referred to above. One only, so far as is known, is patented—that of Dr. R. F. Hunt, of Washington, D. C., which consists of a simple brass oven, large enough to receive the flask, with its screw clamp, and a cover so constructed as to form part of the clamp, and fitting tightly the top of the oven; suitable heating apparatus, a gas jet, alcohol or other lamp being placed under the oven. No thermometer or instrument for registering the temperature is attached, reliance being had on the judgment of the operator, who removes the flask from time to time and tests its temperature by trying the screw and by touching the hot flask with a damp finger, just as the laundress judges of the temperature of her smoothing irons, the "sizzle" indicating the amount of heat present.

Really, while to the careless and unthoughtful, special forms of apparatus and special guards against accident are necessary, with those who are disposed to keep their work well in hand and execute each step thoughtfully and with eyes wide open to results, no special apparatus need be purchased save the flask and screw-clamp. The oven of the kitchen stove or any place where air of a sufficient temperature to soften the plate can be had will answer perfectly well. We have lately had excellent results from closing the flask in a large copper ladle, formerly used to melt lead in, the ladle being set on the gas stove and the heat thus applied. True in this case considerable heat is lost, which in a better constructed apparatus could be economized.

Some, we understand, are using the oil apparatus, minus the oil, with excellent results, an improvement on its original use. No thermometer is used, and indeed it is questionable if this instrument is of much value, for if the operator is present and can see the thermometer, he will be in all probability from time to time trying the screw to see fi the plate is soft—a sufficient guide it would seem. And then too, judging from the fact that of two thermometers possessed by the writer, made to be used with this sort of apparatus, on being immersed in a hot oil bath, the mercury in one stood thirty-five degrees above the other, the conclusion would be reached that the simpler test is as accurate as the more scientific.

Something remains to be said regarding the welding properties of celluloid, and some of its peculiarities, which are deferred until some experiments in progress and in contemplation are completed.

